# Mechanical Evaluation of Frozen and Cryo-Sectioned Papillary Muscle Samples by Using Sinusoidal Analysis: Cross-bridge Kinetics and the Effect of Partial Ca^2+^ activation

**DOI:** 10.21203/rs.3.rs-3516486/v1

**Published:** 2023-11-01

**Authors:** Jing Xi, Han-Zhong Feng, Jian-Ping Jin, Jinxiang Yuan, Masataka Kawai

**Affiliations:** 1 School of Nursing, and Medical Skill Experiment Teaching Center, Suzhou Medical College, Soochow University, Suzhou 215006, China; 2 Dept of Physiology and Biophysics, Univ of Illinois at Chicago, 835 S Wolcot Ave, Chicago, IL 60612, USA; 3 The Collaborative Innovation Center, Jining Medical University, Jining, 272067, China; 4 Department of Anatomy and Cell Biology, College of Medicine, University of Iowa, Iowa City, IA 52242, USA.

## Abstract

The use of frozen and cryo-sectioned cardiac muscle preparations, introduced recently by (Feng & Jin, 2020), offers promising advantages of easy transport and exchange of muscle samples among collaborating laboratories. In this report, we examined integrity of such preparation by studying tension transients in response to sinusoidal length changes and following concomitant amplitude and phase shift in tension time courses at varying frequencies. We used sections with 70 μm thickness, isolated fiber preparations, and studied cross-bridge (CB) kinetics: we activated the preparations with saturating Ca^2+^, and varying concentrations of ATP and phosphate (Pi). Our experiments have demonstrated that this preparation has the normal active tension and elementary steps of the CB cycle. Furthermore, we investigated the effect of Ca^2+^ on the rate constants and found that the rate constant $r_{4}$ of the force generation step is proportionate to [Ca^2+^] when it is <5 μM. This observation suggests that the activation mechanism can be described by a simple second order reaction. As expected, we found that magnitude parameters including tension and stiffness are related to [Ca^2+^] by the Hill equation with cooperativity of 4–5, consistent to the fact that Ca^2+^ activation mechanisms involve cooperative multimolecular interactions. Our results are consistent with a long-held hypothesis that process C (phase 2 of step analysis) represents the CB detachment step, and process B (phase 3) represents the force generation step. In this report, we further found that constant H may also represent work performance step. Our experiments have demonstrated excellent CB kinetics with reduced noise and well-defined two exponentials, which are better than skinned fibers, and easier to handle and study than single myofibrils.

## Introduction:

The exploration of active muscle mechanics and kinetics has undergone a progressive evolution, with a trend towards preparations of increasing simplicity to unravel the intricate workings of muscle function. Early investigations employed intact muscles (Hill, 1938) and intact single fibers (Gordon et al., 1966; Pringle, 1967) that were activated by electrical stimulation. Subsequently, glycerinated (Ruegg & Tregear, 1966; Pringle, 1967) and skinned (Podolsky, 1968; Reuben et al., 1971) fibers were introduced, followed by split fibers (Sugi & Gomi, 1984), and more recently, single myofibrils have been studied (Tesi et al., 2000; Iorga et al., 2012). In skinned fibers to myofibrils, chemicals of experimental interest, such as Ca^2+^, ATP, and Pi, can be perfused across the myofilament space and applied directly to cross-bridges (CBs). The advantage of these preparations is that contractile proteins are packed in the high (physiological) concentrations, hence allosteric cooperativity among participating macromolecules is present and intact. Another advantage is that experiments can be performed in the physiological ionic strength solutions.

In contrast, a simplified system such as solution studies of extracted and reconstituted proteins, and more recently, use of single molecule studies with *in-vitro* motility assays have been carried out. These technics have given enormous impacts in muscle research. However, their story is not complete or sometimes inaccurate for the lack of or diminished cooperative interactions between macromolecules, and experiments must be performed in low ionic strength solutions.

Recently, a technique involving frozen and cryo-sectioned preparations has been introduced by (Feng & Jin, 2020). In this report, our objective is to comprehensively assess the integrity of these preparations using sinusoidal length changes and analyzing concomitant amplitude and phase shift (or elastic and viscous moduli) in force responses. Quite interestingly, our findings reveal a superb frequency response function aligning almost perfectly with the two-time constant model, surpassing the performance of conventional skinned fiber results. Noteworthy is the ease of handling of this preparation, enabling a generation of large force. This investigation contributes to the expanding repertoire of experimental materials for studying active muscle mechanics and kinetics, offering valuable insights into striated muscle functions. The easy transport and exchange of samples of the cryo-sectioned preparations hold a promise of promoting collaborative research across global laboratories and advance our understanding of muscle biomechanics.

## METHODS

### Flash freezing of papillary muscles, and cryo-sectioning

These procedures were described in (Feng & Jin, 2020). In brief, adult C57BL/6 mice were anesthetized with isoflurane, the heart was removed, and left ventricular papillary muscles were isolated and quickly frozen in liquid nitrogen. They were then placed in a 1.7 mL Eppendorf tube for storage at −80°C. In a cryostat set at −20°C, the frozen papillary muscle was sectioned with the thickness of 70 μm and 120–140 μm width. The muscle strips were transferred to a 35 mm cell culture dish in the relaxation solution that contained 50% glycerol at −20°C. The cell culture dish was shipped from Chicago to Iowa City with ice packs and kept in −20°C freezer until used for biomechanical experiments.

### Biomechanical experiments

On the day of experiments, a small bundle was removed and mounted in the experimental apparatus. One end was connected to a linear motor (length driver) and the other end to a tension transducer via stainless-steel wires with a minute amount of nail polish. They were then stretched to just before resting tension was visible, which generally yielded a sarcomere length of 2.1–2.2 μm in myocardium as determined by optical diffraction at longer sarcomere length (Wang et al., 2013a). The thickness of the preparation was 70 μm, and width varied between 50–100 μm as measured under a dissecting microscope. Isometric tension was recorded during full Ca^2+^ activation, which was followed by the ATP study, the phosphate (Pi) study, the Ca^2+^ study, and the rigor study. When tension reached a steady value, sinusoidal analyses (range: 1–100Hz with 13 frequencies at 0.2% amplitude) were carried out. To test the reproducibility, the standard activation was repeated, and if the tension reproducibility was <80%, the data were not used.

### Sinusoidal analysis

This was performed as described (Kawai & Brandt, 1980; Kawai, 2018b), and the complex modulus $Y_{\text{obs}}=Y(f)$ was obtained. It consists of the complex number, hence the name. It contains two components: elastic modulus (EM=RealY(f)) and viscous modulus (VM=ImagY(f)). The frequency dependent complex modulus data were fitted to Eq. 1, which consists of two exponential processes B and C in cardiac fibers (Kawai et al., 1993; Wannenburg et al., 2000) (see also (Shibata et al., 1987)) using our homemade program Dfit4.exe. The purpose of this fitting is to deduce the rate constants 2πb and 2πc of the exponential processes B and C, respectively, in the most rational way.

Process B Process C

(1) $$Y(f)=H-\frac{Bfi}{b+fi}+\frac{Cfi}{c+fi}$$

where $i=\sqrt{-1}$ and H is a constant. b is the characteristic frequency of exponential process B, with B its magnitude (strength, amplitude), c is the characteristic frequency of exponential process C, with C its magnitude, and H is a constant. From this report onward, we will call this H also as a magnitude for a reason that becomes apparent later. Processes B and C are called exponential processes, because their time domain expression consists of exponential functions:

(2) ΔT(t)=[H-Bexp(-2πbt)+Cexp(-2πct)]ΔL

where ΔL is the length change, and ΔT(t) is the tension transient. The algorithm to find b and c, as well as magnitude parameters H,B and C from Y(f) by data fitting has been described (Kawai & Brandt, 1980). In brief, these fitting parameters are found to minimize $\Sigma {\left|Y_{\text{obs}}-Y_{\text{theo}}\right|}^{2}$, where the summation is over the frequencies used, and the theoretical projection $Y_{\text{theo}}$ is calculated based on Eq. 1 at each frequency used for experiments. The complex modulus extrapolated to the infinite frequency (f→∞) is defined by:

$$Y_{\infty }\equiv Y(\infty )=H-B+C$$

This quantity is often called “stiffness” in muscle mechanics literature, which we follow. To normalize available data, magnitudes and related parameters are divided by $Y_{\infty }$ of the nearest standard activation (pCa 4.55, 5 mM MgATP and 8 mM Pi). Thus, the unit of H,B,C and stiffness is $Y_{\infty }$, which is called $Y_{\text{act}}$ and defined in Eq. 6 later.

$Y_{\infty }$ corresponds to phase 1 of step analysis, process C to phase 2, process B to phase 3, and process A to phase 4. Step analysis has been used to study CB kinetics (Huxley & Simmons, 1971; Heinl et al., 1974; Huxley, 1974). Process A has not been observed in cardiac muscles with experiments at ≤25°C (Kawai et al., 1993; Wannenburg et al., 2000), but it appears with experiments at ≥30°C (Lu et al., 2006). When characteristic frequencies are multiplied by 2π, these are called the “apparent rate constants”, signifying experimentally observed rate constants. These are equivalent to the rate constants deduced from tension transients of step analyses, but different from the “intrinsic rate constants” of elementary steps used to describe CB model.

### Standard activation and the rigor study

**Fig. 1.**
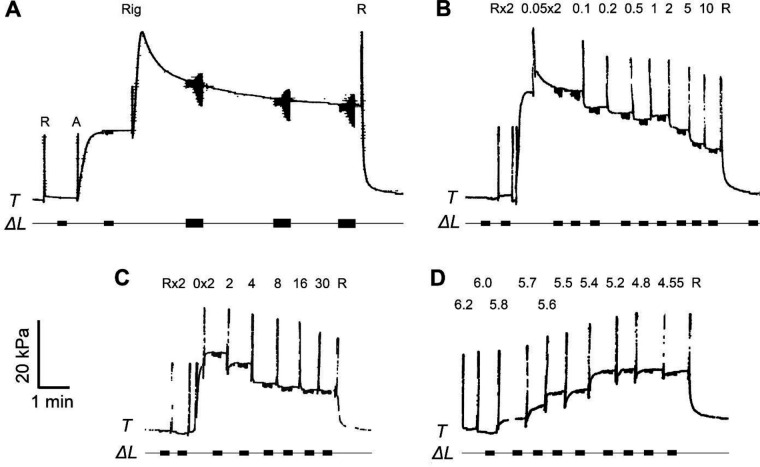
Time course of tension (T) records in slow pen traces. A. Standard activation and rigor study. R=Relaxing solution, A=standard activating solution, Rig=Rigor solution. B. ATP study. The numbers indicate [MgATP] in mM. C. Phosphate (Pi) study. The numbers indicate [Pi] in mM. D. Ca study. The numbers indicate pCa values, where $pCa=-{log}_{10}[{Ca}^{2+}]$. For B and C, x2 indicates that the solution was changed twice. In all panels, computer record of the length change (ΔL) is shown below tension time course. Amplitude was 0.2% $L_{0}$ (peak-to-peak was 0.4% $L_{0}$) for most experiments, except for the rigor condition (amplitude 0.35% $L_{0}$). For the rigor condition, the measurement duration was prolonged, and the amplitude was increased to minimize noise. Tension signal was filtered by 10 Hz second order low pass filter as seen in the rigor record: tension oscillation appears to diminish at higher frequency, because of the filtering. Sharp vertical spikes indicate solution change artifacts. Scale bars represent 20 kPa (ordinate) and 1 min (abscissa).

This was performed as shown in Fig. 1A. The fiber was first relaxed (R), followed by activation (A). Tension rose to reach a plateau. After carrying out sinusoidal analysis quickly (0.4 s/frequency) at low amplitude (0.2% $L_{0}$), the fiber was then transferred to the rigor solution (Rig). Tension further increased, followed by a decrease, taking several minutes to approach a steady state. Two to three more sinusoidal analysis records were taken with a longer duration (1.6 s/frequency) and at an increased amplitude (0.35% $L_{0}$) to obtain the data with better signal-to-noise ratio (S/N). This is because the rigor data were used to correct other experimental data for system response, most of which is caused by the tension transducer, as described earlier (Kawai & Brandt, 1980). In addition, the fiber run down is minimal after induction of the rigor state compared to the Ca^2+^ activated state(s), hence not much is lost by prolonging the data collection period or increasing the oscillation amplitude. In contrast, fiber run down is much more severe when the fiber is activated with Ca^2+^ in the presence of ATP. This part of experiments was usually carried out after the ATP and the Pi studies.

### ATP study and analysis of the apparent rate constant 2πc

The ATP study was carried out as in Fig. 1B for the slow pen trace of the tension time course. Fibers were initially in the relaxing solution (R), and the solution was subsequently changed twice with the one that contained 0.05 mM MgATP in the presence of Ca^2+^ ([Ca^2+^]=22~45 μM, or pCa=4.65~4.35). Because the relaxing solution contained 2 mM MgATP, the first solution change was used to wash out excess MgATP. Tension quickly developed to reach a plateau. The fiber length was then oscillated in a series of sinewaves ranging 1 Hz and 100 Hz. Time course data from both the length (ΔL) and the force (ΔF) changes were collected in every 10 μs, the signal was averaged on each sinusoidal cycle, and the complex modulus data Y(f) were deduced as described (Kawai & Brandt, 1980). Y(f) was then fitted to Eq. 1 to find the apparent rate constants, 2πb and 2πc. After one or two sets of such analyses, [MgATP] was increased to the next level (0.1, 0.2, 0.5, 1, 2, 5 and 10 mM) and the sinusoidal analysis was repeated. After challenging with 8 different MgATP concentrations, fibers were relaxed in R (Fig. 1B). These experiments were carried out in the presence of the ATP regenerating system (15 mM CP and 80 unit/ml CK), thus contaminating ADP was <20 μM (Kushmerick et al., 1992) and negligible. In the ATP study, [Pi] was kept constant at 8 mM, and pCa was kept at 4.55 ([Ca^2+^]=28 μM). The experiments were also carried out in the decreasing order of [ATP] in the next preparation, and the results were averaged to minimize the effect of fiber run down.

### Phosphate (Pi) study and analysis of the apparent rate constant 2πb

The Pi study was carried out as in Fig. 1C. To deduce the kinetic constants ($r_{4},r_{-4},K_{5}$) of the elementary steps surrounding force generation (step 4 in Fig. 4) and phosphate (Pi) release (step 5), P=[Pi] was changed such as 0, 2, 4, 8, 16 and 30 mM. They represent added concentrations of Pi to the experimental saline. Pi was included in the saline as the equimolar mixture of KH_2_PO_4_ and K_2_HPO_4_ (Table 1), and P represents the total Pi concentration. The actual Pi concentration was likely to be 0.7–0.8 mM higher than these added concentrations because of continuous ATP hydrolysis by the fibers and diffusion of Pi to the surrounding saline (Kawai & Halvorson, 1991; Dantzig et al., 1992). The apparent rate constant 2πb was then studied as the function of [Pi]. 2πb reflects the force generation and Pi release steps (Kawai & Halvorson, 1991; Kawai et al., 1993). Fibers were initially relaxed in the relaxing solution (R), then the solution was changed twice with one that does not contain Pi (0 P solution). Because the relaxing solution contained 8 mM Pi, the first solution was used to wash out excess Pi. Tension quickly rose to reach a plateau (Fig. 1C). The fiber length was then oscillated to carry out sinusoidal analysis at 13 frequencies. [Pi] was then increased to the next level, and the experiment was repeated. After sequentially challenging the fibers with six different Pi concentrations, fibers were relaxed with the relaxing solution, R. In the Pi study, [MgATP] was kept constant at 5 mM, and pCa was kept at 4.55. The experiments were also carried out in the decreasing order of [Pi] in the next preparation, and the results were averaged to minimize run down artifact.

### Ca^2+^ study

This was performed at pCa 7.0, 6.2, 6.0, 5.8, 5.7, 5.6, 5.5, 5.4, 5.2, 4.8, and 4.55 as in Fig. 1D (see also Fig. 1 of (Zhang et al., 2021)), where $pCa\equiv -{log}_{10}\left[{Ca}^{2+}\right]$, and isometric tension was recorded as reported (Lu et al., 2010; Zhang et al., 2021). [MgATP] was kept at 5 mM and [Pi] at 8 mM. At each pCa solution, sinusoidal analysis was performed, and the complex modulus was recorded. After the maximum [Ca^2+^], the fiber was relaxed with R.

### Solutions

This is listed in Table 1, much of which been reported (Wang et al., 2013b; Wang et al., 2014). In brief, the relaxing solution contained 10 mM EGTA, 2.4 mM ATP, 4 mM MgAc_2_, and 8 Pi. Activating solutions contained 6 mM CaEGTA, 0–12 mM ATP, 1.7–12 mM MgAc_2_, and 0–30 mM Pi. All activating solutions contained the ATP regenerating system: 15 mM Na_2_CP (creatine phosphate) and 80 unit/ml CK (creatine kinase). For the pCa study, the ratio [Na_2_CaEGTA]:[Na_2_K_2_EGTA] was adjusted to achieve the desired pCa value by keeping the total [EGTA] constant (6 mM) and by using our homemade computer program, ME.exe (multiple equilibria). The rigor solution contained (mM:) 6 mM CaEGTA, 1.55 mM MgAc_2_, and 8 mM Pi. In all solutions, [Mg^2+^]=1 mM, the ionic strength was 200 mM, and pH was adjusted to 7.00 by KOH. All experiments were carried out at 25°C.

### Statistical analysis

All data are expressed as the mean ± standard error (SEM).

## RESULTS

### Cross-bridge (CB) kinetics during standard activation

**Fig. 2.**
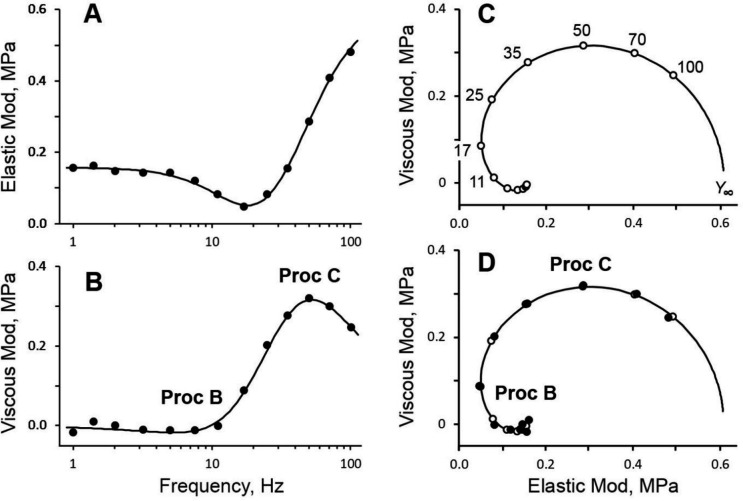
Plots of complex moduli of standard activation. A. Elastic modulus *vs.* frequency. B. Viscous modulus *vs*. frequency. C and D. Elastic *vs.* viscous moduli (Nyquist plot), where each filled circle represents data point at a particular frequency. In A, B and D, filled circles represent the experimental data. Curved lines (and open circles in C and D) are the theoretical values based on Eq. 1 and best fit parameters. In C, theoretical projections based on Eq. 1 (curved line) with frequencies used for measurements (open circles). Frequency points are indicated in Hz in C, except for 7.5, 5, 3.2, 2, 1.4, and 1 Hz (counter-clockwise direction), which are not indicated. $Y_{\infty }$ is on the elastic modulus axis at which point the complex modulus extrapolates for f→∞. D superimposes C and experimentally observed data points shown in A and B. Many open circles (theory) overlap with solid circles (measurements), hence not all are visible. The fitting procedure minimized the sum of squares of their distances. Note that the viscous modulus becomes slightly negative for frequencies <10Hz (B, C and D), hence the abscissa has been lowered by 0.05 MPa. In B and D, approximate locations of processes B and C (Proc B, Proc C) are indicated. The data are based on a single set of measurement and not averaged. The coefficient of correlation of this fitting was 0.998. The unit of moduli is MPa, and the unit of frequency is Hz.

This is depicted in Fig. 1A. Fibers were initially soaked in the relaxing solution (R), and transferred to the activating solution (A). When the steady tension developed, the length of the fibers was oscillated with a small amplitude $\left(0.2\%L_{0}\right)$. This length change corresponded to ~1 nm/CB with 50% series compliance. Concomitant change in tension was recorded by a computer with an Advantech PCA-6743F CPU with homemade interface (Kawai, 2018a) and analyzed in terms of discrete Fourier transform as reported (Kawai & Brandt, 1980; Kawai, 2018b; Kawai et al., 2021). The complex modulus (=Y(f)) were recorded as functions of frequency (f). The results are plotted in Fig. 2. The elastic modulus (EM≡RealY(f)) has a minimum at 17 Hz (Fig. 2A). This frequency is close to the one called “$f_{min}$“, which is defined as the frequency that gives the minimum value on the dynamic modulus |Y(f)| (Shibata et al., 1987). The EM is very large at the high frequency end (100 Hz) because CBs do not have enough time to adjust to the imposed length change, hence it registers a momently frozen structure. The EM is small at the low frequency end (1 Hz) because CBs have adequate time to adjust to the imposed length change. The viscous modulus (VM≡ImagY(f); Fig. 2B) peaks at 50 Hz, which approximates the characteristic frequency c. Although difficult to see, the VM has a minimum at 5–7 Hz where VM<0. This frequency approximates the characteristic frequency b. The muscle preparation produces “oscillatory work” at around this frequency (Pringle, 1967; Kawai et al., 1977; Kawai & Brandt, 1980).

The Nyquist plot is a plot of EM in the abscissa *vs.* VM in the ordinate (Fig. 2C and D). This plot exhibits two semicircles, with the large one opening downward (process C), and small one opening upward (process B). One semicircle corresponds to one exponential process, therefore these Nyquist plots demonstrate that there are two exponential processes involved in the length-tension response in activated cardiac muscles and as shown in Eq. 1 (Kawai et al., 1993; Wannenburg et al., 2000). These are called “exponential” processes, because their time-domain expression consists of exponential functions as in Eq. 2 (Kawai & Brandt, 1980). The quality of the exponential time course is very difficult to judge by its appearance, in particular when there are multiple exponentials, whereas in Nyquist plots, these are easy to identify and to judge the quality of the data because of their circular nature (see Fig. 2C).

To find out the apparent rate constants (2πb and 2πc) in the most rational way, the complex modulus data were fitted to Eq. 1 (Kawai & Brandt, 1980; Kawai et al., 1993; Wannenburg et al., 2000). This process is equivalent to fitting the time course data to two exponential functions to deduce two apparent rate constants. The sinusoidal analysis method gives more accurate estimate of the rate constants in a larger frequency (or time) range. In Fig. 2, the best fit theoretical data (calculated by Eq. 1) are expressed in continuous curves and open circles. Fig. 2C is entirely based on Eq. 1 with best fit parameters and frequency points (as indicated) used for experiments. Fig. 2D superimposes the experimental data points on Fig. 2C. The coefficient of correlation of this fitting was 0.998 for a single measurement. This is much better than the data from ordinary single muscle fibers or cardiac muscle strips, because these latter preparations require averaging of many Y(f) data to have a satisfactory fit. As is demonstrated in Fig. 2D, the data points and theoretical points superimpose extremely well. This can be also seen in Fig. 2A and B, indicating the high quality of the data and appropriateness of Eq. 1 to describe the frequency dependent complex modulus data.

### Rigor study

To determine the significance of stiffness of in-series components, the solution was washed with the rigor solution (Rig; Fig. 1A), which did not contain ATP, CP, or CK. Rigor is a condition in which all possible CBs are attached to the thin filament, thus, it is a measure of the stiffness of the overall sarcomere structure. Rigor tension initially increased, then gradually decreased (Fig. 1A). The rigor tension reached steady state in a few minutes, and the complex modulus data were collected. Rigor stiffness at f=100Hz was chosen, because it varies little with frequency when rigor is developed (Saeki et al., 1991; Murphy et al., 1996). Results are shown in Table 2.

### Elementary steps surrounding ATP binding and cross-bridge detachment

**Figure 3.**
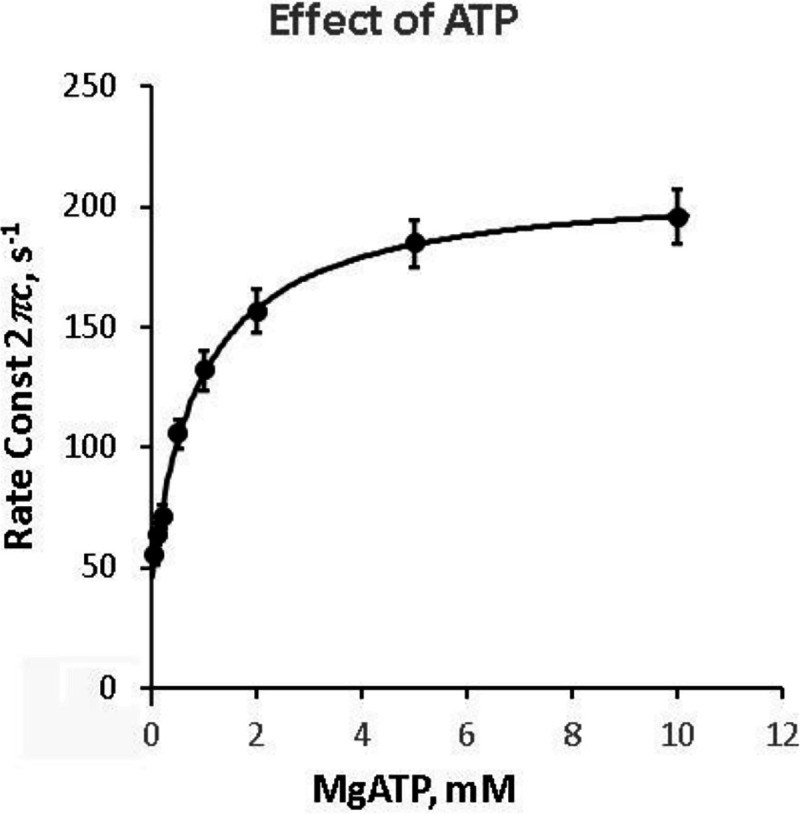
Effect of MgATP on apparent rate constant 2πc. At 8 mM Pi. [MgATP]=0.05,0.1,0.2,0.5,1,2,5,10mM. The mean and SEM are shown. N=18. Files: WT.ATP.R, Mut vs WT 1.xslx. The data were fitted to the three-state model (Eq. 3), that includes step 1 and step 2 in Fig. 4.

**Fig. 4.**
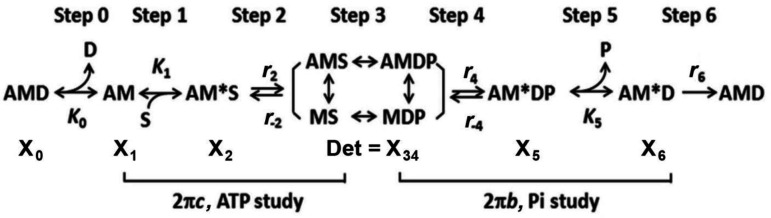
Cross-bridge (CB) model with 6 states. The complex modulus data Y(f) were analyzed based on this CB model, where D=MgADP, S=MgATP, P=Pi=phosphate, A=actin, and M=myosin. Det is an assembly of weakly attached states (AMS and AMDP) and detached states (MS and MDP). $X_{i}$ is the probability of CBs in each state. 2πc represents steps 0–2, and 2πb represents steps 4–5. r’s are the intrinsic rate constants, and K’s are the equilibrium constants (including the association constants) of elementary steps.

The apparent rate constant 2πc reflects the CB detachment step which rapidly ensues the ATP binding step (Kawai, 1978; Kawai & Halvorson, 1989; Kawai et al., 1993). To characterize these steps, the effect of [MgATP] on 2πc was studied in the range 0.05 mM and 10 mM and as shown in Fig. 1B. 2πc increased as S=[MgATP] was increased in sub mM range, and approached saturation thereafter (Fig. 3). These results can be explained by steps 1 and 2 of CB model shown in Fig. 4. Eq. 3 relates 2πc to the MgATP concentration (S=[MgATP]) and the kinetic constants $\left(K_{1},r_{2},r_{-2}\right)$ of elementary steps (Kawai & Halvorson, 1989).

(3) $$2\pi c=\frac{K_{1}S}{1+K_{1}S}r_{2}+r_{-2}$$

The data points are fitted to Eq. 3 by minimizing the sum of squares, from which three kinetic constants ($K_{1},\;r_{2}$, and $r_{-2}$) of the elementary steps are deduced. $K_{1}$ is the MgATP association constant (step 1), $r_{2}$ is the rate constant of the CB detachment step 2, and $r_{-2}$ is the rate constant of its reversal (see Fig. 4). The data (discrete points) fit well to Eq. 3, and as shown in the smooth curve of Fig. 3, demonstrating the high reliability of the CB model. Here it is important to emphasize that we have derived only three parameters ($K_{1},\;r_{2}$, and $r_{-2}$) from the ATP experiment (Fig. 3), and we interpret them in terms of two elementary steps 1 and 2, which is the minimal CB model. Therefore, this is the simplest interpretation of the data. The isometric tension gradually decreased (Fig. 1B) as the [MgATP] was increased and as reported previously (Kawai & Halvorson, 1989; Wang et al., 2014). The results of the ATP study are summarized in Table 3.

### Elementary steps surrounding the force generation and phosphate (Pi) release

**Figure 5.**
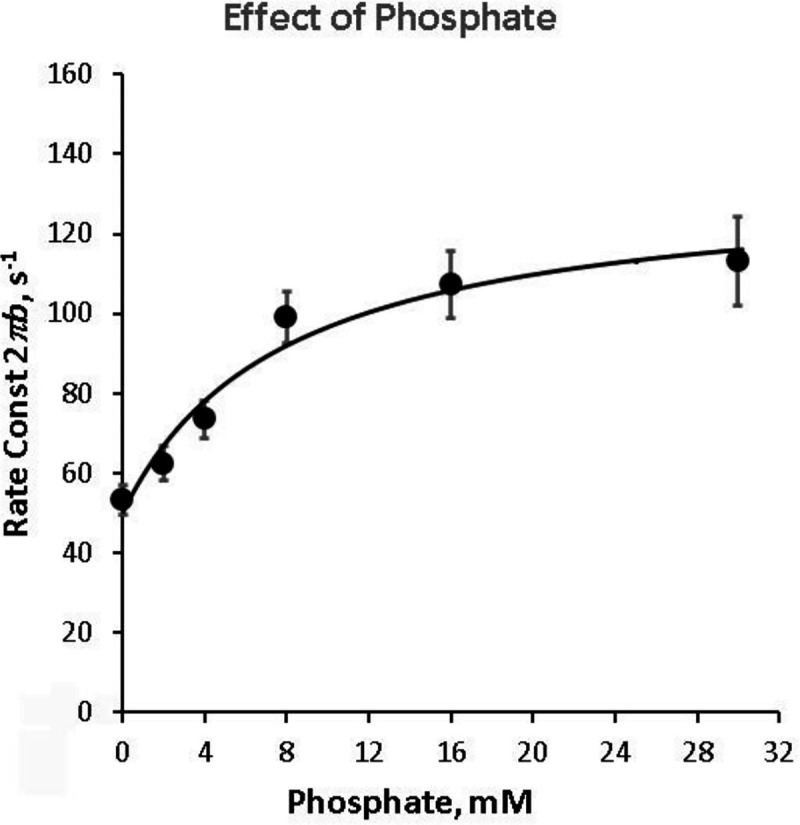
Phosphate effect on the apparent rate constant 2πb. [MgATP]=5mM. The mean and SEM are shown. Average of 18 experiments. The data were fitted to the three-state model (Eq. 4), that includes steps 4 and 5 in Fig. 4.

The apparent rate constant 2πb reflects the force generation step 4 and Pi release step 5 (Fig. 4) (Kawai & Halvorson, 1991; Kawai et al., 1993). To characterize these steps, P=[Pi] was changed in the range 0 and 30 mM, and 2πb was followed. The results are plotted in Fig. 5 (discrete points) as the function of P.2πb increased at low mM Pi, and approached saturation thereafter, exhibiting a hyperbolic saturation curve. Such result can be explained by steps 4 and 5 of the CB model depicted in Fig. 4. The effect of Pi on 2πb is fitted to Eq. 4. This equation is based on steps 4 and 5 of Fig. 4, and relates the Pi concentration (P) and the kinetic constants $\left(r_{4},r_{-4},K_{5}\right)$ of elementary steps 4 and 5 to the apparent rate constant 2πb (Kawai & Halvorson, 1991; Kawai et al., 1993):

(4) $$2\pi b=\sigma r_{4}+\frac{K_{5}P}{1+K_{5}P}r_{-4}$$

where

(5) $$\sigma \equiv \frac{K_{2}K_{1}S}{1+\left(1+K_{2}\right)K_{1}S}$$

σ accounts for the rapid equilibria (steps 1–2, Fig. 4) that exist to the left of step 4. $K_{1}$ and $K_{2}$ obtained from the ATP study with S=5mM (condition of the Pi study) were used to calculate σ from Eq. 5. The data fit well to Eq. 4 as shown in the continuous curve in Fig. 5, demonstrating the high reliability of the CB model in Fig. 4. Once again, it is important to emphasize that we have deduced only three parameters ($r_{4},\;r_{-4}$ and $K_{5}$) from the plot of the Pi dependence of 2πb (Fig. 5), hence Fig. 4 with steps 4 and 5 is the minimal model to account for the data. The isometric tension gradually decreased as the [Pi] was increased (Fig. 1C) and as reported earlier (Kawai, 1986; Kawai & Halvorson, 1991; Kawai et al., 1993; Tesi et al., 2000; Wang et al., 2014). The results of the Pi study are summarized in Table 3.

### Distribution of CBs among 6 states

**Figure 6.**
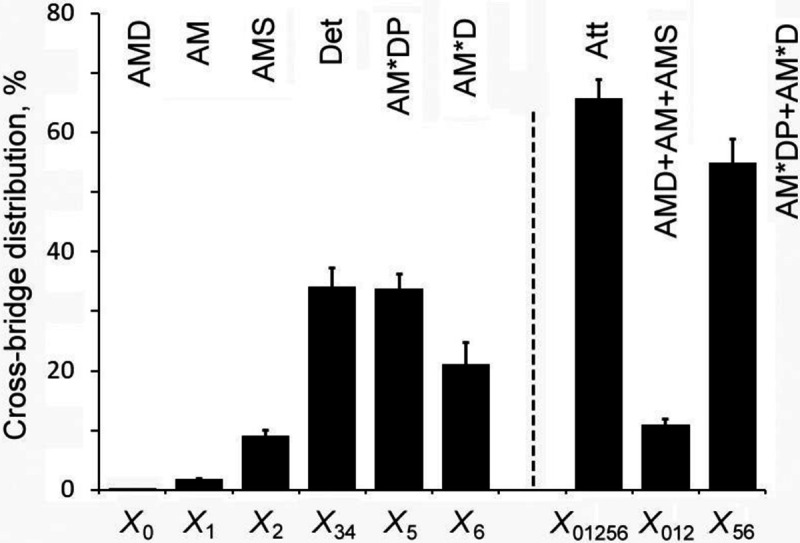
Cross-bridge distribution. Cross-bridge distribution is calculated from equilibrium and association constants in Table 3 at S=5mM and P=8mM based on Eqs. 8–14 of Zhao & Kawai (1996). Errors were propagated from SEM in Table 3. $Att=AMD+AM+AMS+AM*DP+{AM}^{*}D=X_{0}+X_{1}+X_{2}+X_{5}+X_{6}=X_{01256}$. These are force generating, strongly attached states. $X_{34}$ is an assembly of detached and weakly attached states without force, and $X_{01256}+X_{34}=1$. To calculate $X_{0},\;K_{0}\approx 10{\;K}_{1}$ with [MgADP]≈0.01mM were assumed. $X_{0}\approx 0.18\%$.

It is important to know how CBs are distributed among six states in Fig. 4, and how they change depending on S=[MgATP] and P=[Pi], the CB distribution was calculated based on equilibrium constants that includes the association constants thus obtained ($K_{1},\;K_{2},\;K_{4},\;K_{5}$ in Table 3) using Eq. 8–14 published in (Zhao & Kawai, 1996) based on the CB scheme in Fig. 4. Because we have not performed the ADP study from which $K_{0}$ (MgADP association constant) can be determined, $K_{0}∼10K_{1}$ was assumed as the ratio of $K_{0}/K_{1}$ generally resulted in a range between 6–14 in cardiac preparations (Lu et al., 2003; Lu et al., 2005; Wang et al., 2013a; Bai et al., 2014), meaning that ADP binds ~10X more strongly than ATP to the nucleotide binding site of myosin in cardiac muscle fibers. D=[MgADP] in the presence of CP/CK is about 0.01 mM (Kushmerick et al., 1992). The exact values for $K_{0}$ and D do not matter much, however, because $X_{0}=K_{0}DX_{1}$, which is a small value compared to $X_{1}$, which itself is small (Fig. 6). Errors were propagated based on SEM for each kinetic constant. This plot demonstrates that CBs are distributed among the detached states (Det, $X_{34}$: 35%), and in the strongly attached states before (AM*ADP.Pi, $X_{5}$: 34%) and after (AM*ADP, $X_{6}$: 20%) the Pi release step 5 (total is $X_{56}$: 54%). The distribution of CB states in AM.ADP, AM, AM.ATP (total is $X_{012}$: 11%) are small. These are the states that exist after work performance. The force generating or force bearing (strongly attached) states $\left(X_{\text{att}}\right)$ are the summation of $X_{012}$ and $X_{56.}$.

### pCa-tension study and Ca^2+^ sensitivity

**Fig. 7.**
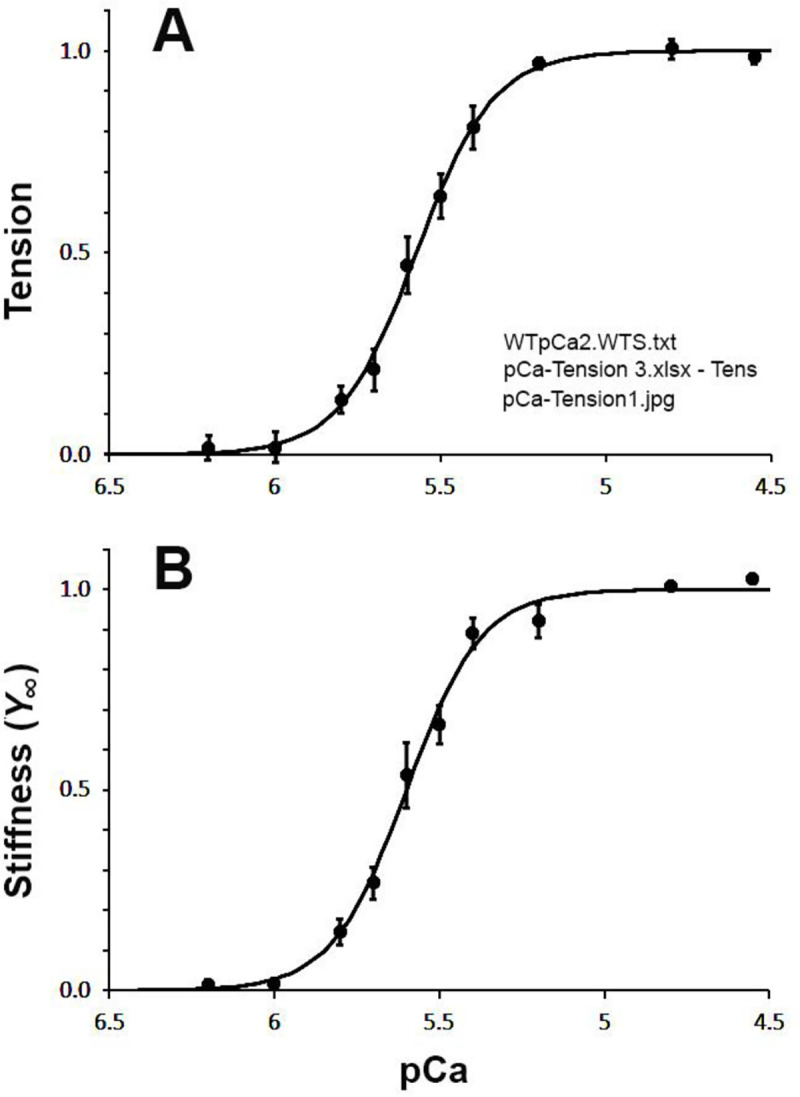
Effect of pCa, where $pCa=-{log}_{10}\left[{Ca}^{2+}\right]$. A on tension, and B on Stiffness $\left(Y_{\infty }\right)$. Each pCa-tension (or stiffness) curve was fitted to Eq. 6 (Eq. 7), followed by a subtraction of $T_{\text{LC}\;}\left(Y_{\text{LC}}\right)$ then the result was divided by $T_{\text{act}\;}\left(Y_{\text{act}}\right)$. The data were then averaged over 13 experiments, and plotted with SEM. Some SEM error bars are smaller than symbol size, and cannot be seen.

This is carried out as described in the Methods section and as depicted in Fig. 1D. The results are plotted as discrete points in Fig. 7A. There is no active tension at pCa 8–6.5 and the muscle is relaxed. Tension increases in a sigmoidal manner as the [Ca^2+^] is increased (pCa<6.5), and it reaches a saturation by pCa 5.3–4.55. The pCa-tension curve was fitted to Hill equation (Eq. 6) (Hill, 1910; Bai et al., 2011) using our homemade program F_pCaTSc.exe:

(6) $$Tension=T_{LC}+\frac{T_{act}}{1+{\left(\frac{Ca_{50}}{\left[Ca^{2+}\right]}\right)}^{n_{H}}}$$

where $T_{\text{LC}}=low\;\left[{Ca}^{2+}\right]$ tension during relaxation for pCa 8–6.5 measured on the chart paper, $T_{\text{act}}$=the amount of tension activated by ${\text{Ca}}^{2+}$, ${\text{Ca}}_{50}=[{\text{Ca}}^{2+}]$ at 50% $T_{\text{act}}$, and $n_{H}$=cooperativity (Hill factor). ${\text{Ca}}_{50}$ is the apparent Ca^2+^ dissociation constant, and ${pCa}_{50}\equiv -{log}_{10}{Ca}_{50}$ is called “Ca sensitivity”. Each pCa-tension curve was first fitted to Eq. 6 to determine $T_{LC},\;T_{\text{act}},\;{Ca}_{50}$ and $n_{H}$ for each experiment. This procedure was followed by subtraction of $T_{LC}$ from tension data, then the result was divided by $T_{\text{act}}$ for normalization. The normalized data were then averaged for multiple pCa-tension measurements and plotted in Fig. 7A with SEM. The averaged fitted parameters are listed in Table 4. The Ca^2+^ sensitivity (${\text{pCa}}_{50}$) was found to be 5.576±0.031 (N=13, SEM), cooperativity ($n_{H}$) 4.57±0.43, and active tension ($T_{\text{act}}$) 14.5±2.3 kPa. The averaged coefficient of correlation was 0.984±0.006. The curved line is the theoretical curve based on Eq. 6 with the best fit parameters. As shown in this Figure, Eq. 6 explains the pCa-tension data well.

### pCa-stiffness results

The pCa-tension data are sometimes scattered, which is mostly caused by the shape of the meniscus of the pin that connects muscle fibers to the tension transducer, and we measure force in the vicinity of 10 μN. In contrast, stiffness $\left(Y_{\infty }\right)$ is less sensitive to the surface tension and sometimes yield better results. For this reason, pCa-stiffness data were processed similarly to the tension data. An equation similar to Eq. 6 was used to fit the stiffness data and to find $Y_{LC},\;Y_{\text{act}},\;Ca_{50Y}$ and $n_{HY}$.

(7) $$\mathrm{Stiffness}=Y_{LC}+\frac{Y_{act}}{1+{\left(\frac{Ca_{50}}{\left[Ca^{2+}\right]}\right)}^{n_{HY}}}$$

This fitting yielded ${\text{pCa}}_{50Y}$ to be 5.584±0.027, $n_{HY}$ 4.90±0.46, $Y_{\text{act}}$ 479±55 kPa, and the coefficient of correlation was 0.991±0.003 (N=13) (Table 4). Consequently, the fitting was slightly better with stiffness than tension. Statistically, ${\text{pCa}}_{50Y}$ and $n_{HY}$ values are not significantly different from respective values of ${\text{pCa}}_{50}$ and $n_{H}$ obtained from the tension data. Fig. 7B is averaged result from the stiffness data and the theoretical curve based on Eq. 7. As can be inferred from these results, Eq.7 fit the stiffness data well, indicating the appropriateness of Eq. 7 for describing the pCa-stiffness data. These results justify use of the pCa-stiffness data to evaluate Ca^2+^ sensitivity and cooperativity. In this report, magnitude parameters $\left(H,B,C,Y_{\infty }\right)$ are normalized (divided) by $Y_{\text{act}}$ of the standard activation, where $Y_{\text{act}}$ is the closest $Y_{\infty }$ at pCa 4.55, 5 mM MgATP and 8 mM Pi, and defined by Eq. 7. Consequently, the unit of magnitude parameters is $Y_{\infty }$.

### CB kinetics as the function of [Ca^2+^]

**Fig. 8.**
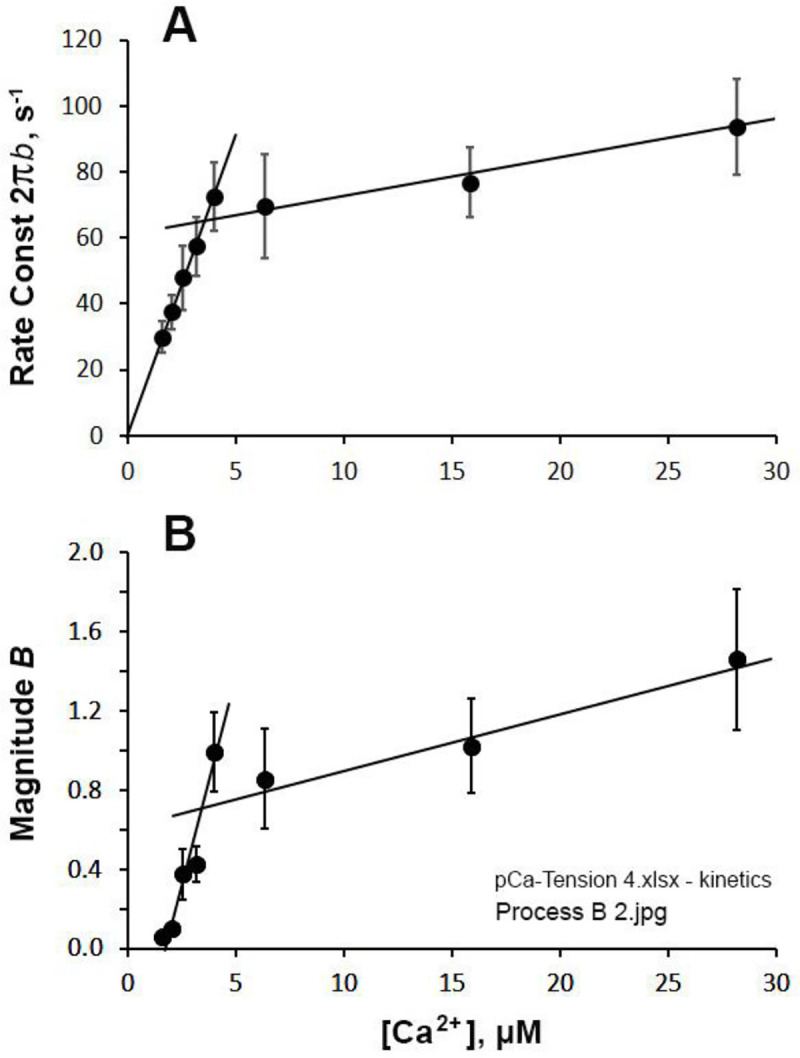
Effect of [Ca^2+^] (linear scale) on exponential process B. A, on the apparent rate constant 2πb, and B, on the magnitude B. Before averaging, B was normalized (divided) by $Y_{\text{act}}$ of the standard activation. Consequently, the unit of B is $Y_{\text{act}}$. Average of 12 measurements with SEM are shown. Lines are drawn by eye to show the approximate trend of the data.

**Fig. 9.**
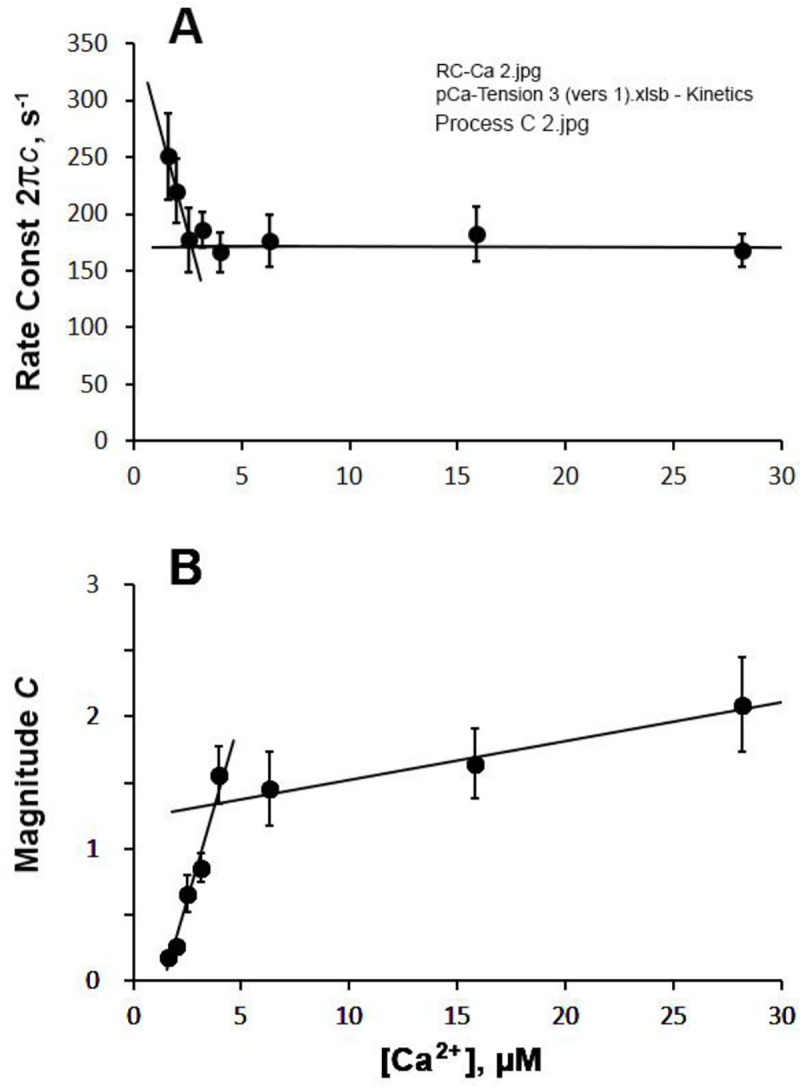
Effect of [Ca^2+^] on exponential process C. A, on 2πc, and B, on magnitude C. The data were similarly treated as in Fig. 8. The unit of C is $Y_{\text{act}}$ Lines are drawn by eye to show the approximate trend.

**Fig. 10.**
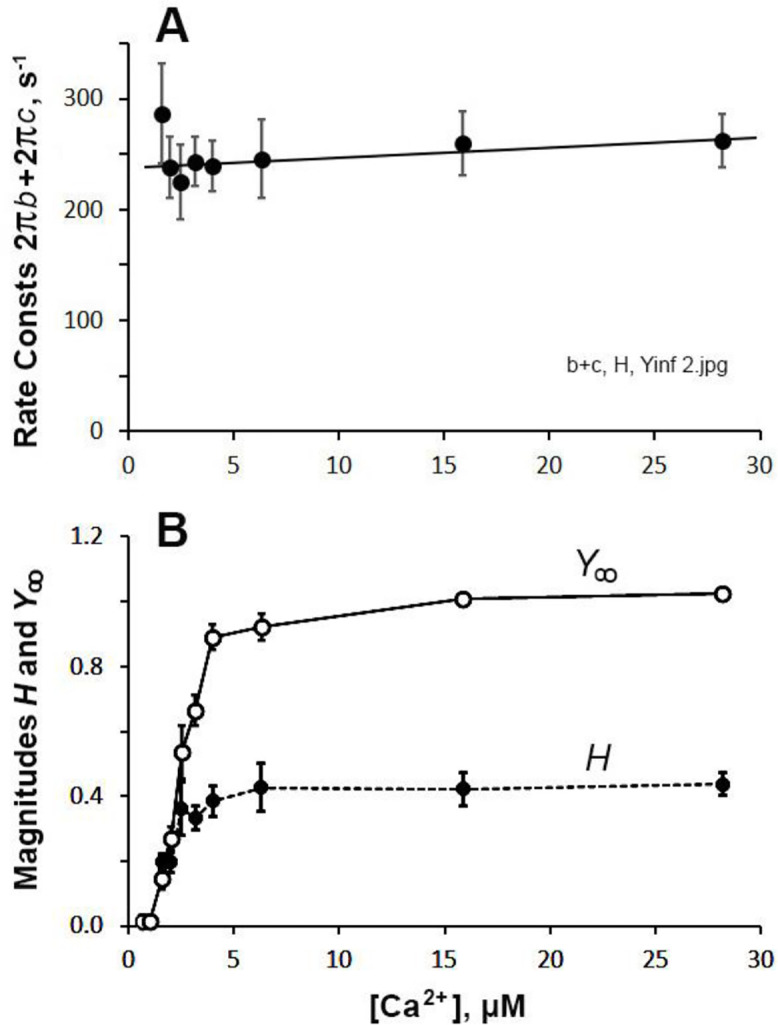
Effect of Ca^2+^ on exponential processes. A, on the sum 2πb+2πc. Line is drawn by eye to show the trend of the data. B, Effect of Ca^2+^ on H and $Y_{\infty }$ with the unit $Y_{\text{act}}$. Lines are drawn to connect data points. $Y_{\infty }$ is the same data as those plotted in Fig. 7B in log (pCa) scale.

We have carried out sinusoidal analysis during partial [Ca^2+^] activations, and the kinetic parameters are summarized in Figs. 8–10. The apparent rate constant 2πb is plotted in Fig. 8A, and 2πc in Fig. 9A in the linear [Ca^2+^] scale in μM. The straight lines are entered by eye to show the trend of the data. The data in Fig. 8A or 9A did not fit to usual hyperbolic saturation equation, because of the sharp inflexion of the data at ~5 μM [Ca^2+^]. This inflexion divides the data in two sections: 5 points for [Ca^2+^] < 5 μM, the rising phase, and 3 points for [Ca^2+^] > 5 μM, the saturation phase. The rising phase of 2πb appears to extrapolate through the origin (Fig. 8A), which may be significant. The magnitude parameter B also had a similar trend as in 2πb (Fig. 8B), but the line of the rising phase did not go through the origin. In principle, B should have a positive (or 0) intercept as [Ca^2+^]→0 (see below). All the magnitude parameters $\left(H,B,C,Y_{\infty }\right)$ are divided, or “normalized”, by $Y_{\text{act}}$ (defined by Eq. 7) of full activation at pCa 4.55, 5 mM MgATP and 8 mM Pi. In another words, the unit of magnitudes is $Y_{\text{act}}$.

The apparent rate constant 2πc (Fig. 9A) decreased for the first 5 points, and saturated for additional 3 points. At the same time, its magnitude C (Fig. 9B) increased steeply at [Ca^2+^] < 5 μM and approached saturation at [Ca^2+^] > 5 μM; this latter effect was similar to magnitude B in Fig. 8B. The sharp increase in 2πb and the decrease in 2πc look to be a mirror image, so their sum 2πb+2πc was calculated and plotted in Fig. 10A. This plot shows that the sum is almost constant, except for the lowest [Ca^2+^] point at 1.58 μM. Fig. 10B plots magnitude H and $Y_{\infty }$. Both plots show the normal saturation curves, except at very low [Ca^2+^]. In principle, these magnitude parameters $\left(H,B,C,Y_{\infty }\right)$ should have zero (0) intercept as [Ca^2+^]→0 (no activation), similar to pCa-tension plot (Fig. 7A), thus plots must be curved for [Ca^2+^] between 0 and 1.58 μM.

## DISCUSSION

### Advantages of frozen and cryo-sectioned preparation for mechanical studies

We have used frozen and cryo-sectioned cardiac muscle preparations to examine its usefulness for muscle mechanical study. We examined integrity of such preparations by studying force transients in response to sinusoidal length changes and following concomitant elastic and viscous responses in force time courses at varying frequencies. We used 70 μm thick and 50–100 μm wide preparations to study cross-bridge kinetics: we activated the preparations with Ca^2+^ and varying concentrations of ATP and phosphate (Pi). Our results demonstrate excellent cross-bridge kinetics with reduced noise and well-defined exponentials, which are better than skinned fibers, and easier to handle than single myofibrils. An advantage of the cryosection method is that the strips are uniform in cross sectional area and uniform perfusion effect for accurate and reproducible mechanical measurements in contrast to the traditional trabeculae preparations. This feature is especially valuable for kinetic studies. This preparation was introduced recently by (Feng & Jin, 2020), which has promising features of muscle preparations of uniform cross sectional area and easy transport for exchange of muscle samples among collaborating laboratories around the world.

The active tension was 16.30±1.08 kPa (Table 2), which is on the low side compared to others. The main reason is because our standard activating solution contained 8 mM Pi with ionic strength adjusted to 200 mM in addition to the presence of 5 mM MgATP (Table 1). These conditions lower the isometric tension for 2–4 folds. The same holds true for other parameters that include stiffness and magnitude parameters. The tension:stiffness ratio and the apparent rate constants do not have this problem, and the values in Table 2 compare well with other preparations reported earlier. Evidently, the rate constants change sensitively depending on the ATP and Pi concentrations, and fit well to the CB model depicted in Fig. 4. When rigor was induced, tension transiently increased and came back with a slow time course (Fig. 1A). The number of attached CBs do not change for stiffness remains the same during the slow decline of tension; thus this decline must be caused by a slippage of CBs to a lesser tension site on actin as observed in strained CBs with in-vitro motility assays (Kawai et al., 2006; Oguchi et al., 2011).

What is different from usual fiber work is that we were able to obtain high quality data that fit well to a simple two exponential process model (Eq. 1 and Fig. 2), in particular, at high frequency range where process C is resident. Consequently, the process C is better defined in the cryo-sectioned preparations than in cardiac muscle strips/fibers. Similar improvement in the quality of process C was observed with single myofibril experiments (Kawai et al., 2021). Evidently, the order of simplification of preparations is: muscle strips > muscle fibers > cryo-sectioned preparations > myofibrils. Thus, gradual improvement of the process C must be related to the simplification of the preparation – which suggests us that the extracellular matrix (ECM) is a major cause of the distortion of process C. The ECM includes sarcolemmal membrane and associated collagen fibers as well as blood vessels and other ECM molecules and structures. It is easy to imagine that these structures interfere with fast process C to make it to appear as a distributed rate constant, because water molecules have to move in and out of ECM elements as the fiber length is oscillated, thus serving as an extra dumping element.

Process C corresponds to phase 2 of step analysis and these two methods observe the same molecular events, or elementary steps in the CB cycle. In fact, a possibility of existence of multiple exponential processes was suggested by several investigating groups. (Abbott & Steiger, 1977) and (Kawai & Zhao, 1993) fitted this phase to two exponentials, while (Ford et al., 1977) fitted this phase to four exponentials, all in muscle fibers. The results from these preparations were probably masked by passive components such as ECM, and as proposed by (Abbott & Steiger, 1977).

### Cross-bridge kinetics and elementary steps of the CB cycle

One positive aspect is that we were able to deduce exactly the same CB model (Fig. 4) whether cryo-sectioned cardiac strips were used or cardiac muscle strips were used, except that there may be small changes in the rate constants (Table 3). This fact confirms the universality of proposed CB model of Fig. 4 and experimentally measured rate constants of elementary steps. Comparing stiffness data during the standard activation (529±36 kPa, Table 2) and in rigor (1010±63 kPa), it can be concluded that about 52±9% (=529/1010) of CBs are made during the standard activation. This number compares well to the distribution of CBs at $X_{56}$ (54±6%, Fig. 6) that includes the AM*ADP.Pi and AM*ADP states and calculated based on the kinetic constants (Table 3) measured in this report. The attached CBs may have to include other strongly attached states AM.ADP, AM and AM.ATP states, but their sum $X_{012}$ is 11±1% (Fig. 6), and do not significantly change the results. What is important here is that the number of attached CBs is not 5–10% as estimated from duty ratio using *in-vitro* motility assay experiments (Harris & Warshaw, 1993; Webb et al., 2013). It is possible that a presence of a tight molecular coupling (called “cooperativity”) in the fiber system may make a difference from the *in-vitro* system. The ionic strength of the activating solution is much less in the *in-vitro* assays (≤50 mM) compared to fiber studies (~200 mM) which may also make a difference.

### Process B and Ca^2+^ activation

Our results demonstrate that whether active tension or stiffness is used, we arrive at the same Ca^2+^ sensitivity and cooperativity results (Fig. 7, Table 4). This fact is convenient when the tension data are not as much reliable as the stiffness data at small force level such as <10 μN, where the shape of the meniscus interfaces with force measurements.

In partial Ca^2+^ activation, it is interesting to note that the rate constant of process B (2πb) decreases (Fig. 8A), whereas that of process C (2πc) increases (Fig. 9A) as [Ca^2+^] is decreased below 5 μM, and the two exponential processes B and C become further apart and more dissociated. This fact implies that processes B and C represent two different molecular steps, and as has been hypothesized (Kawai & Halvorson, 1991). The correlation between process C and the detachment step has been shown from the ATP study on 2πc (such as in Fig. 3), and the correlation between process B and the force generation step has been shown from the Pi study on 2πb (such as in Fig. 5). Our earlier assumption that process B represents the force generation step (Kawai & Halvorson, 1991) is consistent with the result of pressure-release experiment (Fortune et al., 1991) and caged Pi experiment (Dantzig et al., 1992).

What is interesting is that, at low Ca^2+^ activation (at the rising phase of pCa-tension curve), a plot of 2πb-[Ca^2+^] is linear and it appears to go through the origin: 2πb is proportionate to [Ca^2+^] (Fig. 8A). As discussed above, 2πb represents force generation step 4 (Eq. 4, Fig. 4). Its proportionate relationship with [Ca^2+^] at < 5 μM (Fig. 8A) indicates that the Ca^2+^ activation mechanism can be approximated by a second order reaction:

(8) $$r_{4}=r_{40}\left[{Ca}^{2+}\right]$$

This would be an empirical equation to relate the Ca^2+^ activation mechanism to the force generation step; a similar mechanism was initially proposed by (Julian, 1969) as an “activation factor”. For [Ca^2+^] > 5 μM, this activation mechanism approaches a saturation (Fig. 8A). Actual activation starts from Ca^2+^ binding to TnC, and subsequent intermolecular interactions with TnI, TnT, Tpm, actin and myosin, which are tightly coupled and strongly allosteric (cooperative). These in turn result in a steep pCa-tension (or stiffness) relationship (Fig. 7) with the Hill coefficient $\left(n_{H}\right)$ amounting to 4–5 (Table 4). Compared to this high order coupling, the mechanism expressed in Eq. 8 is a very simple relationship between [Ca^2+^] and the rate constant of force generation $\left(r_{4}\right)$ with the Hill factor of 1, and may be useful for modeling the Ca^2+^ activation mechanisms.

The apparent rate constant 2πc represents the CB detachment step 2 (Eq. 3, Figs. 3 & 4) (Kawai & Halvorson, 1989). Because the effect of [Ca^2+^] on 2πc is relatively small (Fig. 9A) compared to that on 2πb (Fig. 8A)*,* we conclude that the major effect of Ca^2+^ is on process B, and its effect on process C may be secondary and can be caused by the interaction with its effect on process B. Thus, we should not state here that [Ca^2+^] affects CB detachment step 2, and that the detachment is lessened as [Ca^2+^] is increased up to 5 μM. The fact, that their sum (2πb+2πc) is not very much variable for the most of [Ca^2+^] studied (Fig. 10A), may suggest that the effect of Ca^2+^ on process C may be indeed secondary. The fact, that 2πc decreases when [Ca^2+^] is at variance with the model proposed by (Huxley & Simmons, 1971; Huxley, 1974) that phase 2 of tension transient in step analysis (process C in sinusoidal analysis) represents the force generation step.

### Magnitude H and the low frequency component

The magnitude parameter H is a constant in Eq. 1, which is the elastic modulus extrapolated to the zero frequency (f→0). In cardiac preparations, such as used in this report, H has a substantial value (Fig. 10B) amounting to 40% of $Y_{\text{act}}$ (Table 2). Furthermore, we found that H is [Ca^2+^] sensitive (Fig. 10B) and becomes activated similar to tension or stiffness, and as in the case of magnitudes B and C (Figs. 8B and 9B). Thus, H must have a molecular origin stemming from the activation mechanism, which is common to all magnitude parameters ($H,\;B,\;C,\;Y_{\infty }$, and tension). This situation is quite different from that of fast twitch skeletal muscle fibers in which H is a small constant and no meaning has been assigned to it.

Magnitude H is a low frequency component, and it likely includes process A observed in fast twitch skeletal fibers (Kawai et al., 1977; Kawai & Brandt, 1980). While process A is absent in experiments with cardiac preparations performed at ≤25°C, the process A emerges at higher temperatures (≥30°C) (Lu et al., 2006). It is likely that H includes the effect of process A when this is not visible. The process A correlates well with the ATP hydrolysis rate, hence it may represent the step 6 in Fig. 4 with the rate constant $r_{6}$, which is the slowest step in the CB cycle (Kawai & Zhao, 1993; Wang & Kawai, 2013). Because force is generated at step 4 before Pi release (step 5), and step 6 is slower than steps 4 and 5, step 6 is the likely place where work performance takes place. A generation of movement requires a transfer of momentum, which takes substantial time (Kawai & Zhao, 1993).

## Conclusions

We conclude that magnitude C represents the number of CBs going through detachment (step 2), magnitude B represents the number of CBs performing force generation (step 4), and magnitude H represents CBs performing work (step 6). $Y_{\infty }$ represents strongly attached CBs, and active tension is the linear sum of the strongly attached states (Kawai & Zhao, 1993). These magnitude parameters are primarily related with CB numbers in each state or transition between states. Their levels are activated by Ca^2+^ in a cooperative manner to result in the large Hill factor of 4–5. The effect of Ca^2+^ on the rate constant of the force generation step can be approximated by a second order reaction (Eq. 8). We found that usefulness of frozen and cryo-sectioned cardiac muscle preparations, and that we obtained excellent kinetic data with many meaningful insights into the molecular mechanisms of contraction.

## Figures and Tables

**Table 1.** Solutions used in this report (mM)

Solution name	Relaxing	Activating	Rigor	MgATP study		Pi study	
Symbol	R	A	Rig	0S	10S	0P	30P

K_2_CaEGTA	-	6	6	6	6	6	6
K_2_H_2_EGTA	10	-	-	-	-	-	-
Na_2_H_2_ATP	2.4	6.06	-	-	12.1	6.12	6.06
Na_2_CP	-	15	-	15	15	15	15
MgAc_2_	3.9	6.61	1.55	1.69	11.53	6.68	6.44
KH_2_PO_4_	4	4	4	4	4	0	15
K_2_HPO_4_	4	4	4	4	4	0	15
KAc	65	53.5	88.7	74.0	33.2	72.1	14.5
KCl	12	12	12	12	12	12	12
NaAc	20	12.8	55	25	0.8	12.8	0.9
MOPS	10	10	10	10	10	10	10
CK (unit/ml)	0	80	0	80	80	80	80

CP=creatine phosphate. CK=Creatine kinase. S=[MgATP], $P=[\text{Pi}]=[{\text{PO}}_{4}{]}_{\text{total}}$. mS indicates mM concentration (m) of the solutions of the ATP study. Intermediate S solutions are made by an appropriate mixture of 0S and 10S solutions: nS=[n(10S)+(10-n)(0S)]/10 by volume. nP indicates mM concentration (n) of the solutions of the Pi study. Intermediate P solutions are made by an appropriate mixture of 0P and 30P solutions: nP=[n(30P)+(30-n)(0P)]/30 by volume. A is the standard activating solution, which is the same as 5S and 8P solutions. In all solutions, pH was adjusted to 7.00 by KOH. $[{\text{Mg}}^{2+}]=1\;\text{mM}$, $[\text{Na}{]}_{\text{total}}=55\;\text{mM}$. The ionic strength was 200 mM.

**Table 2.** Parameters from the standard activation and rigor at 25°C. The activating solution contained 5 mM MgATP, 8 mM Pi, and ionic strength was 200 mM. pCa 4.55 and pH 7.00.

Parameter	Average ± SEM	unit	# observations

*Standard activation*			
Tension	16.30 ± 1.08	kPa	42
Stiffness $Y_{\infty }$	529 ± 36	kPa	42
Tension:Stiffness	3.21 ± 0.14	%$L_{0}$	42
Rate Const 2πb	80.3 ± 5.5	s^−1^	42
Rate Const 2πc	181 ± 9	s^−1^	42
Magnitude H	211 ± 15	kPa	42
Magnitude B	487 ± 55	kPa	42
Magnitude C	806 ± 67	kPa	42
*Rigor*			
Tension	21.67 ± 1.48	kPa	39
Stiffness at 100Hz	1010 ± 63	kPa	39
Tension:stiffness	2.18 ± 0.10	%$L_{0}$	39

**Table 3.** Kinetic constants of elementary steps at 25°C

Kinetic constants	Average ± SEM	unit	# observations

$K_{1}$, ATP association constant	1.03 ± 0.09	mM^−1^	20
$r_{2}$, CB detachment RC	162.5 ± 10.0	s^−1^	20
$r_{-2}$, Reverse detachment RC	48.3 ± 3.8	s^−1^	20
$K_{2}$, CB detachment EC	3.74 ± 0.32	-	20
$r_{4}$, Force generation RC	64.5 ± 3.7	s^−1^	14
$r_{-4}$, Reverse force generation RC	87.1 ± 12.9	s^−1^	14
$K_{4}$, Force generation EC	0.988 ± 0.157	−	14
$K_{5}$, Pi association constant	0.200 ± 0.042	mM^−1^	14

RC=rate constant, EC=equilibrium constant

**Table 4.** Ca^2^+ sensitivity study at 25°C (5 mM MgATP, 8 mM Pi, 200 mM IS)

Parameter	Average ± SEM	# of curves

*Based on Tension*		
${\text{pCa}}_{50}$, Ca^2^+ sensitivity	5.576 ± 0.031	13
$n_{H}$, Cooperativity	4.57 ± 0.43	13
$T_{\text{act}}$, Maximum tension	14.5 ± 2.3 kPa	13
cc, coefficient of correlation	0.984 ± 0.006	13
*Based on stiffness*		
${\text{pCa}}_{50Y}$, Ca^2+^ sensitivity	5.584 ± 0.027	13
$n_{\text{HY}}$, Cooperativity	4.90 ± 0.46	13
$Y_{\text{act}}$, Maximum stiffness	479 ± 55 kPa	13
cc, coefficient of correlation	0.991 ± 0.003	13

The data were fitted to Eq. 6 (Tension) or Eq. 7 (Stiffness) by using our homemade program F_pCaTS.exe for both tension and stiffness. No significant difference in ${\text{pCa}}_{50}$ or $n_{\text{H}}$ values whether the tension or stiffness data were used. Total number of data points: 100 for each of tension and stiffness.

## LIST OF SYMBOLS AND ABBREVIATIONS USED

A: Magnitude of process A
Ac: Acetate
ADP: Adenosine-5’-diphosphate
AM: Actomyosin, actin and myosin
ATP: Adenosine-5’-triphosphate
B: Magnitude of process B
b: Characteristic frequency of process B. 2πb is its apparent (measured) rate constant
C: Magnitude of process C
c: Characteristic frequency of process C. 2πc is its apparent rate constant
${\text{Ca}}_{50}$: Apparent Ca dissociation constant, based on pCa-tension plot
${\text{Ca}}_{50Y}$: Apparent Ca dissociation constant, based on pCa-stiffness plot
CB: Cross-bridge
CK: Creatine kinase
CM: Complex modulus
CP: Creatine phosphate, phosphocreatine
D: =[MgADP], concentration of MgADP
DM: Dynamicmodulus=/Y(f)|
EGTA: Ethylene glycol-bis(β-aminoethyl ether)-N,N,N′,N′-tetraacetic acid
EM
f: Frequency used for sinusoidal analysis
H: Complex modulus at f→0, and may include process A
i: $i=\sqrt{-1}$, imaginary number
$K_{0}$: CB’s MgADP association constant
$K_{1}$: CB’s MgATP association constant
$K_{2}$: The equilibrium constant of CB detachment step 2
$K_{4}$: The equilibrium constant of force generation step 4
$K_{5}$: CB’s phosphate (Pi) association constant. $1/K_{5}$ is Pi dissociation constant
$l_{0}$: Length of muscle preparation
$n_{H}\;$: Cooperativity (Hill factor) measured from pCa-tension plot
$n_{HY}$: Cooperativity (Hill factor) measured from pCa-stiffness plot
P: P=[Pi]=[phosphate], phosphate concentration
π: Circumference ratio, π=3.14159265…
pCa: $=-{\text{log}}_{10}[{\text{Ca}}^{2+}]$
${\text{pCa}}_{50}$: $=-{\text{log}}_{10}[{\text{Ca}}_{50}]$, calcium sensitivity measured from pCa-tension curve
${\text{pCa}}_{50Y}$: $=-{\text{log}}_{10}[{\text{Ca}}_{50Y}]$, calcium sensitivity measured from pCa-stiffness curve
PH: Phaseshift=arg[Y(f)]
Pi: Phosphate
$r_{2}$: Rate constant of CB detachment step 2
$r_{-2}$: Rate constant of CB’s reverse detachment step 2
$r_{4}\;$: Rate constant of force generation step 4
$r_{-4}$: Rate constant of reverse force generation step 4
$r_{6}\;$: Rate constant of work performance step 6
S: Substrate concentration, S=[MgATP]
σ: See Eq. 5 for definition
$S_{\text{act}}$: Ca activatable stiffness
$S_{\text{LC}}$: Low Ca^2+^ stiffness
$T_{\text{act}}$: Ca activatable tension
$T_{\text{LC}}$: Low Ca^2+^ tension
VM: Viscousmodulus=Imag[Y(f)]
$X_{0}$: Probability of CBs in the AM.ADP state
$X_{1}$: Probability of CBs in the AM state
$X_{2}$: Probability of CBs in the AM.ATP state
$X_{012}$: Probability of strongly attached CBs after work performance. $X_{012}=X_{0}+X_{1}+X_{2}$
$X_{34}$: Probability of CBs in the detached and weakly attached states
$X_{5}$: Probability of CBs in the AM*ADP.Pi state
$X_{6}$: Probability of CBs in the AM*ADP state
$X_{56}$: Probability of CBs after force generation, but before work performance, $X_{56}=X_{5}+X_{6}$
$X_{01256}$: Probability of CBs in the strongly attached states. $X_{01256}=X_{0}+X_{1}+X_{2}+X_{5}+X_{6}=1-X_{34}$
$Y_{\infty }\;$: Stiffness (Complex modulus extrapolated to the infinite frequency, f→∞)
Y(f): Complex modulus
$Y_{\text{act}}\;$: Ca activatable stiffness
$Y_{\text{LC}}$: Low Ca^2+^ stiffness

## References

[R1] Abbott RH & Steiger GJ. (1977). Temperature and amplitude dependence of tension transients in glycerinated skeletal and insect fibrillar muscle. J Physiol 266, 13–42. 856995 10.1113/jphysiol.1977.sp011754 PMC1283551

[R2] Bai F, Caster HM, Rubenstein PA, Dawson JF & Kawai M. (2014). Using baculovirus/insect cell expressed recombinant actin to study the molecular pathogenesis of HCM caused by actin mutation A331P. J Mol Cell Cardiol 74C, 64–75. 10.1016/j.yjmcc.2014.04.014 PMC4264970 24793351

[R3] Bai F, Weis A, Takeda AK, Chase PB & Kawai M. (2011). Enhanced active cross-bridges during diastole: molecular pathogenesis of tropomyosin’s HCM mutations. Biophys J 100, 1014–1023. 21320446 10.1016/j.bpj.2011.01.001 PMC3037557

[R4] Dantzig J, Goldman Y, Millar NC, Lacktis J & Homsher E. (1992). Reversal of the cross-bridge force-generating transition by the photogeneration of phosphate in rabbit psoas muscle fibers. J Physiol 451, 247–278. 1403812 10.1113/jphysiol.1992.sp019163 PMC1176160

[R5] Feng HZ & Jin JP. (2020). High efficiency preparation of skinned mouse cardiac muscle strips from cryosections for contractility studies. Exp Physiol 105, 1869–1881. 32857888 10.1113/EP088521 PMC7914300

[R6] Ford LE, Huxley AF & Simmons RM. (1977). Tension responses to sudden length change in stimulated frog muscle fibres near slack length. J Physiol 269, 441–515. 302333 10.1113/jphysiol.1977.sp011911 PMC1283722

[R7] Fortune NS, Geeves MA & Ranatunga KW. (1991). Tension responses to rapid pressure release in glycerinated rabbit muscle fibers. Proc Natl Acad Sci (USA) 88, 7323–7327. 1871140 10.1073/pnas.88.16.7323 PMC52287

[R8] Harris DE & Warshaw DM. (1993). Smooth and skeletal muscle actin are mechanically indistinguishable in the in vitro motility assay. Circ Res 72, 219–224. 8417844 10.1161/01.res.72.1.219

[R9] Heinl P, Kuhn HJ & Ruegg JC. (1974). Tension responses to quick length changes of glycerinated skeletal muscle fibres from the frog and tortoise. J Physiol 237, 243–258. 4545181 10.1113/jphysiol.1974.sp010480 PMC1350882

[R10] Hill AV. (1910). The possible effects of the aggregation of the molecules of haemoglobin on its dissociation curves J Physiol (Lond) 40 (Suppl), 4–7.

[R11] Huxley AF. (1974). Muscular contraction. J Physiol 243, 1–43. 4449057 PMC1330687

[R12] Huxley AF & Simmons RM. (1971). Proposed mechanism of force generation in striated muscle. Nature 233, 533–538. 4939977 10.1038/233533a0

[R13] Julian FJ. (1969). Activation in a skeletal muscle contraction model with a modification for insect fibrillar muscle. Biophys J 9, 547–570. 5778185 10.1016/S0006-3495(69)86403-9 PMC1367536

[R14] Kawai M. (1978). Head rotation or dissociation? A study of exponential rate processes in chemically skinned rabbit muscle fibers when MgATP concentration is changed. Biophys J 22, 97–103. 638228 10.1016/S0006-3495(78)85473-3 PMC1473409

[R15] Kawai M. (1986). The role of orthophosphate in crossbridge kinetics in chemically skinned rabbit psoas fibres as detected with sinusoidal and step length alterations. J Muscle Res Cell Motil 7, 421–434. 3491834 10.1007/BF01753585

[R16] Kawai M. (2018a). Biomechanics, Muscle Fibers, and How to Interface Experimental Apparatus to a Computer (Textbook). . Springer International Publishing AG (London, UK).

[R17] Kawai M. (2018b). Mathematics needed to solve problems of contraction. Biomechanics, Muscle Fibers, and How to Interface Experimental Apparatus to a Computer (Textbook), 65–76. Springer International Publishing AG (London, UK).

[R18] Kawai M, Brandt P & Orentlicher M. (1977). Dependence of energy transduction in intact skeletal muscles on the time in tension. Biophys J 18, 161–172. 140712 10.1016/S0006-3495(77)85605-1 PMC1473282

[R19] Kawai M & Brandt PW. (1980). Sinusoidal analysis: a high resolution method for correlating biochemical reactions with physiological processes in activated skeletal muscles of rabbit, frog and crayfish. J Muscle Res Cell Mot 1, 279–303. 10.1007/BF00711932 6971874

[R20] Kawai M & Halvorson H. (1989). Role of MgATP and MgADP in the crossbridge kinetics in chemically skinned rabbit psoas fibers. Study of a fast exponential process C. . Biophys J 55, 595–603. 2785822 10.1016/S0006-3495(89)82857-7 PMC1330542

[R21] Kawai M & Halvorson HR. (1991). Two step mechanism of phosphate release and the mechanism of force generation in chemically skinned fibers of rabbit psoas. Biophys J 59, 329–342. 2009356 10.1016/S0006-3495(91)82227-5 PMC1281150

[R22] Kawai M, Kido T, Vogel M, Fink RH & Ishiwata S. (2006). Temperature change does not affect force between regulated actin filaments and heavy meromyosin in single-molecule experiments. J Physiol 574, 877–887. 16709631 10.1113/jphysiol.2006.111708 PMC1817734

[R23] Kawai M, Saeki Y & Zhao Y. (1993). Cross-bridge scheme and the kinetic constants of elementary steps deduced from chemically skinned papillary and trabecular muscles of the ferret. Circ Res 73, 35–50. 8508533 10.1161/01.res.73.1.35

[R24] Kawai M, Stehle R, Pfitzer G & Iorga B. (2021). Phosphate has dual roles in cross-bridge kinetics in rabbit psoas single myofibrils. J Gen Physiol 153. 10.1085/jgp.202012755 PMC7885270 33599680

[R25] Kawai M & Zhao Y. (1993). Cross-bridge scheme and force per cross-bridge state in skinned rabbit psoas muscle fibers. Biophys J 65, 638–651. 8218893 10.1016/S0006-3495(93)81109-3 PMC1225766

[R26] Kushmerick MJ, Moerland TS & Wiseman RW. (1992). Mammalian skeletal muscle fibers distinguished by contents of phosphocreatine, ATP, and Pi. Proc Natl Acad Sci U S A 89, 7521–7525. 1502163 10.1073/pnas.89.16.7521 PMC49742

[R27] Lu X, Bryant MK, Bryan KE, Rubenstein PA & Kawai M. (2005). Role of the N-terminal negative charges of actin in force generation and cross-bridge kinetics in reconstituted bovine cardiac muscle fibres. J Physiol 564, 65–82. 15649975 10.1113/jphysiol.2004.078055 PMC1456038

[R28] Lu X, Heeley DH, Smillie LB & Kawai M. (2010). The role of tropomyosin isoforms and phosphorylation in force generation in thin-filament reconstituted bovine cardiac muscle fibres. J Muscle Res Cell Motil 31, 93–109. 20559861 10.1007/s10974-010-9213-x PMC3089900

[R29] Lu X, Tobacman LS & Kawai M. (2003). Effects of tropomyosin internal deletion Delta23Tm on isometric tension and the cross-bridge kinetics in bovine myocardium. J Physiol 553, 457–471. 14500764 10.1113/jphysiol.2003.053694 PMC2343557

[R30] Lu X, Tobacman LS & Kawai M. (2006). Temperature-dependence of isometric tension and cross-bridge kinetics of cardiac muscle fibers reconstituted with a tropomyosin internal deletion mutant. Biophys J 91, 4230–4240. 16980359 10.1529/biophysj.106.084608 PMC1635655

[R31] Murphy KP, Zhao Y & Kawai M. (1996). Molecular forces involved in force generation during skeletal muscle contraction. J Exp Biol 199, 2565–2571. 9110950 10.1242/jeb.199.12.2565

[R32] Oguchi Y, Ishizuka J, Hitchcock-DeGregori SE, Ishiwata S & Kawai M. (2011). The role of tropomyosin domains in cooperative activation of the actin-myosin interaction. J Mol Biol 414, 667–680. 22041451 10.1016/j.jmb.2011.10.026 PMC3230701

[R33] Pringle JW. (1967). The contractile mechanism of insect fibrillar muscle. Prog Biophys Mol Biol 17, 1–60. 4226124 10.1016/0079-6107(67)90003-x

[R34] Ruegg JC & Tregear RT. (1966). Mechanical factors affecting the ATPase activity of glycerol-extracted insect fibrillar flight muscle. Proc R Soc Lond B Biol Sci 165, 497–512. 4380573 10.1098/rspb.1966.0080

[R35] Saeki Y, Kawai M & Zhao Y. (1991). Comparison of crossbridge dynamics between intact and skinned myocardium from ferret right ventricles. Circ Res 68, 772–781. 1742866 10.1161/01.res.68.3.772

[R36] Shibata T, Hunter WC & Sagawa K. (1987). Dynamic stiffness of barium-contractured cardiac muscles with different speeds of contraction. Circ Res 60, 770–779. 2954719 10.1161/01.res.60.5.770

[R37] Tesi C, Colomo F, Nencini S, Pirodi N & Poggesi C. (2000). The effect of inorganic phosphate on force generation in single myofibrils from rabbit skeletal muscle. Biophy J 78, 3081–3092. 10.1016/S0006-3495(00)76845-7 PMC1300890 10827985

[R38] Wang L & Kawai M. (2013). A re-interpretation of the rate of tension redevelopment (kTR) in active muscle. J Muscle Res Cell Motil 34, 407–415. 24162314 10.1007/s10974-013-9366-5 PMC3909470

[R39] Wang L, Muthu P, Szczesna-Cordary D & Kawai M. (2013a). Characterizations of myosin essential light chain’s N-terminal truncation mutant Delta43 in transgenic mouse papillary muscles by using tension transients in response to sinusoidal length alterations. J Muscle Res Cell Motil 34, 93–105. 23397074 10.1007/s10974-013-9337-x PMC3656599

[R40] Wang L, Muthu P, Szczesna-Cordary D & Kawai M. (2013b). Diversity and Similarity of Motor Function and Cross-Bridge Kinetics in Papillary Muscles of Transgenic Mice Carrying Myosin Regulatory Light Chain Mutations D166Vand R58Q. J Mol Cell Cardiol 62, 153–163. 23727233 10.1016/j.yjmcc.2013.05.012 PMC3809071

[R41] Wang L, Sadayappan S & Kawai M. (2014). Cardiac myosin binding protein C phosphorylation affects cross-bridge cycle’s elementary steps in a site-specific manner. Plos One 0113417, 1–21. 10.1371/journal.pone.0113417 PMC4242647 25420047

[R42] Wannenburg T, Heijne GH, Geerdink JH, Van-Den-Dool HW, Janssen PM & DeTombe PP. (2000). Cross-bridge kinetics in rat myocardium: effect of sarcomere length and calcium activation. Am J Physiol 279, H779–H790. 10.1152/ajpheart.2000.279.2.H779 10924078

[R43] Webb M, Jackson del R Jr, ., Stewart TJ, Dugan SP, Carter MS, Cremo CR& Baker JE. (2013). The myosin duty ratio tunes the calcium sensitivity and cooperative activation of the thin filament. Biochemistry 52, 6437–6444. 23947752 10.1021/bi400262h PMC7207222

[R44] Zhang J, Wang L, Kazmierczak K, Yun H, Szczesna-Cordary D & Kawai M. (2021). Hypertrophic cardiomyopathy associated E22K mutation in myosin regulatory light chain decreases calcium-activated tension and stiffness and reduces myofilament Ca(2+) sensitivity. Febs j. 10.1111/febs.15753 PMC8668163 33548158

[R45] Zhao Y & Kawai M. (1996). Inotropic agent EMD 53998 weakens nucleotide and phosphate binding to cross bridges in porcine myocardium. Am J Physiol 271 (Heart Circ Physiol 40), H1394–H1406. 8897933 10.1152/ajpheart.1996.271.4.H1394

